# *Daphne odora* Exerts Depigmenting Effects via Inhibiting CREB/MITF and Activating AKT/ERK-Signaling Pathways

**DOI:** 10.3390/cimb44080228

**Published:** 2022-07-22

**Authors:** Young Sic Eom, Dongho Jeong, A-Reum Ryu, Keon-Hyoung Song, Dai Sig Im, Mi-Young Lee

**Affiliations:** 1 Department of Medical Science, Soonchunhyang University, 22 Soonchunhyang-ro, Asan 31538, Chungnam, Korea; 1319yse@sch.ac.kr (Y.S.E.); jeongdh372814@gmail.com (D.J.); beophyen@sch.ac.kr (K.-H.S.); 2 Department of Medical Biotechnology, Soonchunhyang University, 22 Soonchunhyang-ro, Asan 31538, Chungnam, Korea; yar4310@sch.ac.kr; 3 Department of Pharmaceutical Engineering, Soonchunhyang University, 22 Soonchunhyang-ro, Asan 31538, Chungnam, Korea; 4 Department of SC Major on New Medicinal Materials, Division of Student Corporation, Soonchunhyang University, 22 Soonchunhyang-ro, Asan 31538, Chungnam, Korea; dsim@sch.ac.kr

**Keywords:** *Daphne adora*, depigmenting agent, anti-melanogenesis, CREB, MITF, AKT, ERK

## Abstract

*Daphne odora*, a blooming shrub, has been traditionally used for various medicinal purposes. However, information on its anti-melanogenic activity and dermal application is limited. In this study, the *Daphne odora* extract (DOE), with constituents including daphnetin, was used to investigate depigmenting activity and the underlying mechanism of *Daphne odora*. DOE inhibited in vitro and cellular tyrosinase activity in a dose-dependent manner, and reduced the α-MSH-induced melanin biosynthesis to a control level. The protein expressions of melanin synthesis-related enzymes were also significantly reduced by DOE. Moreover, DOE decreased the phosphorylation of cAMP-response element binding proteins (CREBs) induced by α-MSH in B16F10 cells, while it activated phosphorylated extra-cellular signal-regulated kinases (ERKs) and protein kinase B (AKT) expression. These results suggest that DOE might inhibit the melanogenesis signaling pathways by activating ERK- and AKT-signaling pathways to regulate the expression of CREB and MITF and its downstream pathways. Therefore, DOE could potentially be developed as a depigmenting agent.

## 1. Introduction

The complex process of melanin biosynthesis is regulated by tyrosinase- and tyrosinaserelated protein-1 and -2 (TRP-1 and TRP-2) [1]. Tyrosinase is responsible for the first step of melanin production by the hydroxylation of tyrosine into dihydroxyphenylalanine (DOPA) that is subsequently oxidized to DOPA Quinone [2]. DOPA Quinone is non-enzymatically oxidized to DOPA chrome, which is then spontaneously converted to 5, 6-dihydroxyindole (DHI) and 5, 6-dihydroxyindole-2-carboxylic acid (DHICA) [3]. Further oxidation and eventual polymerization results in melanin biosynthesis [4]. Tyrosinase catalyzes the rate-limiting step in melanogenesis, and the inhibition of tyrosinase is the most efficient strategy for the inhibition of melanogenesis and the development of skin hypopigmentation agents [5]. Thus, a variety of inhibitors that target tyrosinase have been studied. During melanogenesis, TRP-2 catalyzes the conversion of DOPA chrome to DHICA, while TRP-1 catalyzes the oxidation of DHICA to eumelanin [6]. TRP-1 and TRP-2, which stabilize and increase tyrosinase activity, are regulated by the microphthalmia-associated transcription factor (MITF), a major transcription factor in melanogenesis [7].

The binding of α-MSH to the melanocortin 1 receptor (MC1R) elicits the activation of adenylate cyclase and the subsequent elevation of the intracellular concentration of cAMP [8]. cAMP leads to the translocation of protein kinase A (PKA) into the nucleus, thus activating phosphorylated cAMP-response element-binding proteins (CREBs) and stimulating the expression of MITF [3]. MITF is regulated by several signaling pathways, including mitogen-activated protein kinases (MAPKs). MAPKs comprise three subfamilies: extra-cellular signal-regulated kinases (ERKs), c-Jun N-terminal kinases (JNKs), and p38-MAPKs [9]. The activation of the p38-MAPK pathway is involved in enhanced melanogenesis, whereas the activation of ERK signaling down-regulates melanogenesis via tyrosinase inhibition. Additionally, the activation of protein kinase B (AKT) signaling is also known to play a pivotal role in melanogenic inhibition [10].

A wide variety of chemicals with a hypopigmentation effect have been developed as skin-brightening agents [11]. They include arbutin, ethyl ascorbylether, ascorbyl glucoside, magnesium ascorbyl phosphate, niacinamide, alpha-bisabolol, and ascorbyl tetraisopalmitate. However, due to the potential side effects and cellular toxicity reported for some of these chemicals, more research has been focused on the development of anti-melanogenic agents using natural extracts with lower side effects [12]. The natural products derived from *Broussonetia kazinoki* [13], *Glycyrrhiza glabra* [14], *Prunus persica* Flos [15], *Phellodendron amurense* [16], *Cudrania tricuspidata* Bureau [17], *Zanthoxylum schinifolium* [18], and *Dendropanax morbifera* [19] have been well studied.

*Daphne odora* is an evergreen shrub that belongs to the red bean flower family. The flower has a very fragrant and strong odor derived from volatile components, including p-phellandrene, nonanal, and geranial. The roots of *Daphne odora* have been used for medicinal purposes such as hemostasis, pertussis, dementia, detoxification, and bruises [20]. The bark and leaves have been used to treat hemorrhoids, disinfection, swelling, poisoning, and the aftermath of a common cold [21]. However, information pertaining to its anti-melanogenetic activity and the results of dermal application is limited. In this study, the anti-melanogenic effect of *Daphne odora* extract (DOE) was examined for the first time, focusing on its inhibitory effect on the signaling pathway of melanin biosynthesis. The inhibitory effect of DOE on the expression of tyrosinase, TRP-1, TRP-2, and CREB/MITF via the activation of the AKT/ERK signaling pathway was analyzed.

## 2. Materials and Methods

### 2.1. Plant Extract and B16F10 Cell Culture

A methanolic extract from the leaf and stem parts of *Daphne odora* used in this study was obtained from the Korea Plant Extract Bank at the Korea Research Institute of Bioscience and Biotechnology (Daejeon, Korea) and diluted to a 2% solution using dimethyl sulfoxide (DMSO). The mouse melanoma cell line, B16F10, was obtained from the Korean Cell Line Bank (Seoul, Korea). Dulbecco’s modified eagle’s media (DMEM), supplemented with 10% fetal bovine serum and 1% penicillin/streptomycin, was used for B16F10 cell culture.

### 2.2. Daphnetin Analysis

Daphnetin (7,8-Dihydroxycoumarin, MW 178.14, purity >97%) and analytical grade trifluoroacetic acid (TFA) were purchased from Sigma (St. Louis, MO, USA). Acetonitrile of HPLC grade was purchased from J. T. Baker (Phillipsburg, NJ, USA). HPLC-grade water was doubly purified with an Arium mini ultrapure water system (Sartorius, Goettingen, Germany) system. All other reagents were of the highest grade commercially available.

### 2.3. LC-UV and MS Analysis

The HPLC system (Prominence series, Shimadzu, Kyoto, Japan) consisted of a degasser, a binary pump (LC-20AD), an autosampler (SIL-20A), a diode array detector, and LabSolutions software. The injection volume onto the LC column was 10 μL of aliquots. Daphnetin was separated on a C18 column (2.1 × 150 mm, 5 μm particle size, Vydac, Hichrom, UK) at 40 °C under the flow (0.2 mL/min) of a mobile phase with gradient elution. The eluents contained water with 0.1% TFA (Eluent A) and acetonitrile with 0.1% TFA (Eluent B). The gradient increased from 7% B for 1 min to 30% B over 2.5 min, followed by 2.5 min at 30% B, decreased to 7% over 0.5 min, and re-equilibrated at 7% B for 3.5 min. UV data were collected at 325 nm.

Mass spectra were recorded on a single quadrupole mass spectrometer system including an electrospray ionization source (LCMS-2020, Shimadzu, Japan) in the positive ion mode. The mass spectrometer was calibrated using a Tune Mix solution (Shimadzu, Japan). Nitrogen (>99.997%) was used as a drying and nebulizing gas. The MS instrument operated in a full-scan mode of 50~200 *m*/*z* with the LabSolutions software under the following MS tuning conditions: detector voltage of 1.25 kV; interface voltage of 4.5 kV; interface temperature of 350 °C; heat block temperature of 200 °C; DL temperature of 250 °C; nebulizing gas flow of 1.5 L/min; and drying gas flow of 15 L/min.

### 2.4. MTT Assay

B16F10 melanoma cells were plated at 1 × 10^4^ cells/well in a 96-well plate and cultured for 24 h. The indicated doses of DOE were added to the cells, and the cells were cultured in a CO_2_ incubator for 12 h. The MTT stock solution was dissolved in phosphate-buffered saline (PBS) and added to the culture medium for 1 h. Then, the culture supernatant was removed, and 200 μL of DMSO was added to each well to dissolve the MTT formazan crystals produced by the surviving cells. Absorbance was measured at 540 nm using a Tecan’s Sunrise absorbance microplate reader.

### 2.5. Inhibition of In Vitro and Cellular Tyrosinase Activity

The tyrosinase reaction mixture consisted of a 1.5 mM L-tyrosine solution and 2000 U/mL mushroom tyrosinase (Sigma), in 0.1 M phosphate buffer (pH 6.8). The sample solution was added to its action mixture and incubated at 37 °C for 10 min. The optical density was measured at 490 nm using a spectrophotometer. The IC_50_ value of the sample was expressed as the concentration of inhibitor at which it inhibited 50% of the tyrosinase activity. The percent inhibition of the tyrosinase reaction was calculated as follows: 

Inhibition (%) = (A − B)/A × 100;

A = Absorbance at 475 nm without test sample after incubation;

B = Absorbance at 475 nm with test sample after incubation.

To measure cellular tyrosinase activity, melanoma cells were treated with the indicated concentrations of DOE (0.98, 1.95, 3.91, and 7.81 μg/mL) together with α-MSH (100 nM) for 24 h. The cells were then harvested and lysed with 1% Triton X-100 buffer. The cell extracts reacted with L-tyrosine substrate (1.5 mM), and then the relative tyrosinase activities were calculated by measuring the optical density (472 nm).

### 2.6. Measurement of Melanin Content

B16F10 cells were seeded in a 6-well plate (1 × 10^5^ cells/well) and then incubated for 72 h. The cells were washed with PBS, and then treated with various concentrations of sample in the presence of 100 nmol/L of α-MSH. After incubation for 3 days, the supernatant was centrifuged at 3000 rpm for 3 min. The supernatant was transferred to 96-well plate and the absorbance was measured at 450 nm with a Tecan’s Sunrise absorbance microplate reader.

### 2.7. Western Blot Analysis

B16F10 cells were cultured in the absence or presence of DOE (0.98, 1.95, or 3.91 μg/mL) for 12 h at 37 °C. Cells were lysed using lysis buffer (50 mM Tris-HCl pH 8.0, 0.1% SDS, 150 mM NaCl, 1% NP-40, 0.02% sodium azide, 0.5% sodium deoxycholate, 100 g/mL PMSF, and 1 g/mL aprotinin), and sonication was used to dissolve the cell pellet. Total proteins were obtained by centrifugation at 12,000 rpm for 40 min at 4 °C. Samples containing 20 μg of total protein were separated by sodium dodecyl sulfate-polyacrylamide gel electrophoresis (SDS-PAGE). The gels were transferred onto activated polyvinylidene difluoride (PVDF) membranes for 1 h at 400 mA. The PVDF membranes were blocked for 2 h in a blocking solution (5% bovine serum albumin or 5% skim milk) at room temperature. The membranes were then incubated for 18 h at 4 °C with the individual primary antibodies against MITF, CREB, p-CREB, pAKT, AKT, pERK, and ERK (1:1000 dilution; Cell Signaling Technology, Danvers, MA, USA). TRP1, TRP2, tyrosinase (1:1000; Santa Cruz Biotechnology, Dallas, TX, USA), and ß-actin (1:5000; Santa Cruz Biotechnology, TX, USA) were also used. The membranes were then incubated with horse-radish peroxidase (HRP)-conjugated secondary antibody diluted 1:5000 for 50 min at room temperature. The secondary antibodies for MITF, CREB, p-CREB, pAKT, AKT, pERK, ERK, and tyrosinase were anti-rabbit IgG, HRP-linked antibodies (Cell Signaling Technology, MA, USA), and for TRP1, TRP2 and ß-actin were anti-mouse IgG, HRP-linked antibodies (Cell Signaling Technology, MA, USA). The secondary antibody for tyrosinase was anti-goat IgG-HRP (Santa Cruz Biotechnology, TX, USA).

Each membrane was visualized using the Supernova ECL Western blotting detection system (Cyanagen s.r.l., Bologna, Italy), and the band images were taken using Sensi-Q 2000 (Lugen Sci Co. Ltd., Bucheon-si, Gyeonggi-do, Korea). ImageJ software was used to quantify protein band intensities.

### 2.8. Statistical Analysis

The data of three independent experiments were statistically analyzed using the Statistical Package for Social Sciences (SPSS, version 20; SPSS Inc., Chicago, IL, USA) and expressed as the mean ± SD of the values for each group. The statistical significance of differences between control and test groups was assessed using one-way analysis of variance (ANOVA) followed by Tukey’s test. *p* < 0.05 was considered statistically significant.

## 3. Results 

### 3.1. In Vitro and Cellular Tyrosinase Activity Inhibition by DOE

**Figure 1:**
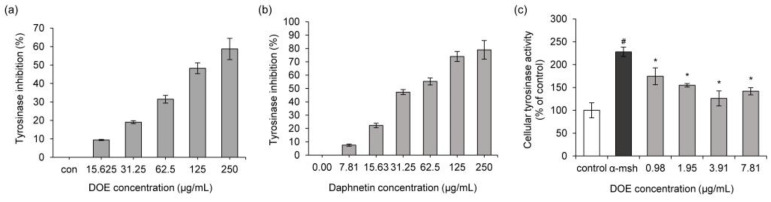
(**a**) *Daphne odora* extract (DOE) inhibits mushroom tyrosinase activity in a dose-dependent manner. (**b**) Daphnetin inhibits mushroom tyrosinase activity in a dose-dependent manner. (**c**) DOE inhibits cellular tyrosinase activity in α-MSH-treated B16F10 cells. ^#^ *p* < 0.05 vs. control. * *p* < 0.05 vs. α-MSH-treated group.

To ascertain whether DOE inhibits the catalytic activity of tyrosinase, various concentrations of the extract were added to a mixture containing mushroom tyrosinase and tyrosine, and then the enzyme’s activity was assayed (Figure 1a). The DOE concentrations of 62.5 and 250 μg/mL inhibited 34.9% and 61.4% of tyrosinase activity, respectively. The approximate IC_50_ value of DOE on the tyrosinase activity was determined to be 137.9 μg/mL. Next, daphnetin, from *Daphne odora*, was tested to measure its tyrosinase inhibitory activity. As expected, at concentrations of 62.5 μg/mL and 250 μg/mL, daphnetin inhibited tyrosinase by approximately 52.1 and 74.9%, respectively (Figure 1b). Thus, the tyrosinase-inhibitory activity of DOE may be due to the presence of phytochemicals, including daphnetin. 

To obtain the inhibiting potency of DOE in the cellular model, the inhibitory effect of DOE on the tyrosinase activity of B16F10 cells treated with α-MSH was measured. Upon treating α-MSH alone, tyrosinase activity significantly increased compared to the untreated control (Figure 1c). However, cellular tyrosinase activity was significantly reduced at 0.98, 1.95, 3.91, and 7.81 μg/mL of DOE. 

### 3.2. Analysis of Daphnetin Content in DOE

**Figure 2:**
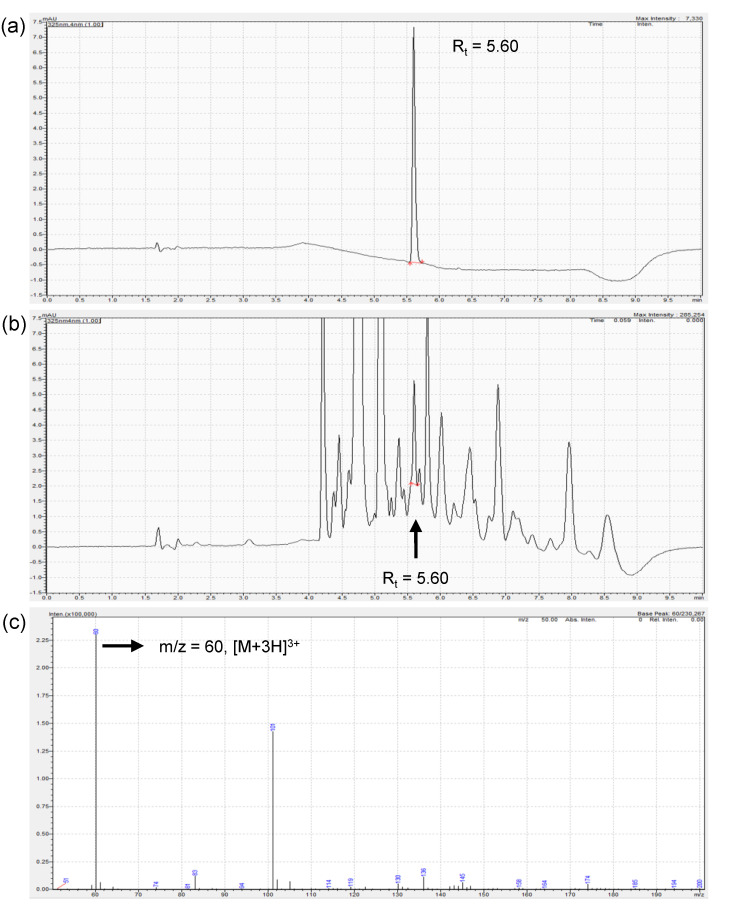
LC-UV chromatography and mass chromatography of DOE showing the presence of daphnetin. (**a**) LC-UV chromatogram of daphnetin. (**b**) LC-UV chromatogram of DOE. (**c**) Mass chromatogram of 5.6 min fraction of DOE from (**b**), corresponding to [M + 3H]^3+^ of daphnetin.

To investigate the presence and content of daphnetin in DOE, LC-UV chromatography and mass spectrum chromatography of DOE were performed, as shown in Figure 2. Daphnetin in DOE was identified at 5.6 min, identical to the corresponding standard daphnetin. The peak fraction of 5.6 min from DOE showed a mass-to-charge ratio, *m*/*z*, of 60, which corresponded to the [M + 3H]^3+^ of daphnetin. The content of daphnetin in DOE was determined to be 0.047%.

### 3.3. Anti-Melanogenic Activity of DOE in B16F10 Cells

**Figure 3:**
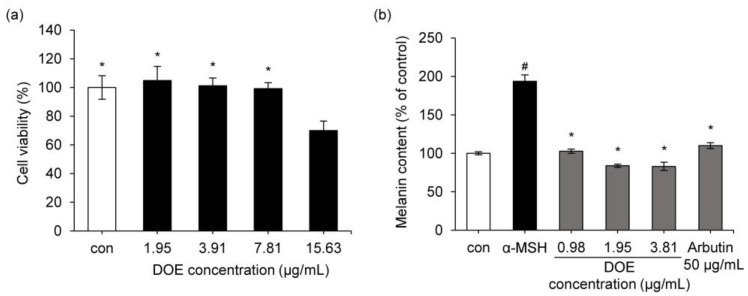
(**a**) Effect of DOE on the viability of B16F10 cells. * *p* < 0.05 vs. DOE 15.63 μg/mL treated group. (**b**) Effect of DOE on α-MSH-induced melanin biosynthesis in B16F10 cells. * *p* < 0.05 vs. α-MSH-treated group. ^#^ *p* < 0.05 vs. control group. Positive control = 50 μg/mL arbutin. Data are presented as mean ± SD.

To rule out the possibility that the anti-melanogenic activity of the extract may be due to its cytotoxic activity and to determine whether DOE had a cytotoxic effect in B16F10 cells, an MTT assay was performed (Figure 3). B16F10 cells were treated with various concentrations of DOE ranging from 1.95 to 15.63 μg/mL for 24 h. As shown in Figure 3a, DOE was not cytotoxic between 1.95 μg/mL and 7.81 μg/mL in melanoma cells.

Figure 3b shows the effects of DOE on melanin biosynthesis in B16F10 cells. The intracellular melanin content increased to 193.8% following α-MSH treatment. However, in the group treated with α-MSH hormone and DOE (0.98 to 3.91 μg/mL) simultaneously, melanin synthesis reduced to the level in the control group (Figure 3b). This result indicates that DOE significantly inhibits melanin biosynthesis in B16F10 cells. Arbutin (50 μg/mL), used as a positive control, inhibited α-MSH-enhanced melanin content to the control level.

### 3.4. Anti-Melanogenic Activity of DOE via Inhibiting CREB/MITF Signaling Pathway

**Figure 4:**
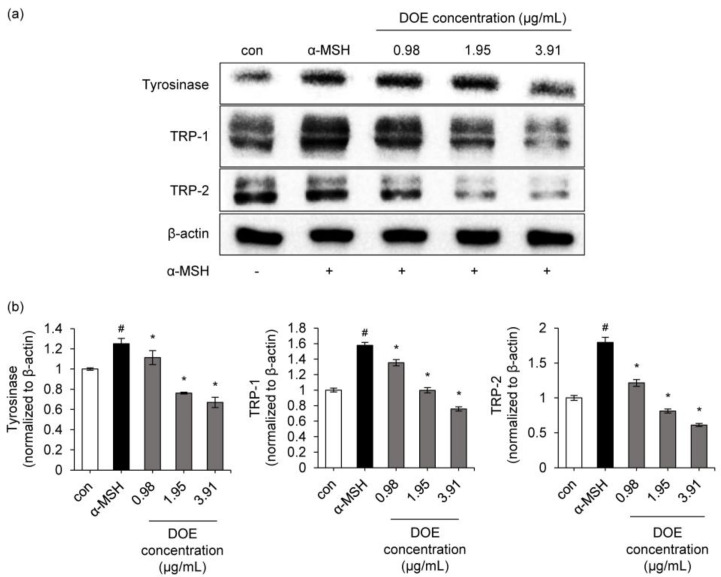
(**a**) Effect of DOE on the protein expressions of tyrosinase, tyrosinase-related protein-1 (TRP-1), and TRP-2 in B16F10 cells. B16F10 cells were treated with different concentrations of DOE ranging from 0.98 to 3.91 μg/mL. Protein expressions of tyrosinase, TRP-1, and TRP-2 were analyzed by Western blotting. Equal protein loading was confirmed using β-actin antibody. (**b**) Protein level of tyrosinase, TRP-1, and TRP-2. The relative level of each protein was calculated based on the intensity of actin protein. ^#^ *p* < 0.05 vs. control. * *p* < 0.05 vs. α-MSH-treated group.

To determine whether the suppressive activity of the extract on melanin contents is associated with the level of melanogenesis-related proteins, such as TRP-1, TRP-2, and tyrosinase, Western blotting was conducted using cells exposed to α-MSH in either the absence or presence of the extract. As shown in Figure 4, tyrosinase, TRP-1, and TRP-2 protein levels in the B16F10 cells treated with DOE were reduced in a dose-dependent manner. A DOE concentration of 3.91 μg/mL inhibited α-MSH-induced expression of tyrosinase, TRP-1, and TRP-2 by 47%, 55%, and 66%, respectively, compared to the α-MSH-treated group. These results suggest that the inhibition of melanogenesis by DOE is associated with the down-regulation of melanogenesis signaling pathways.

**Figure 5:**
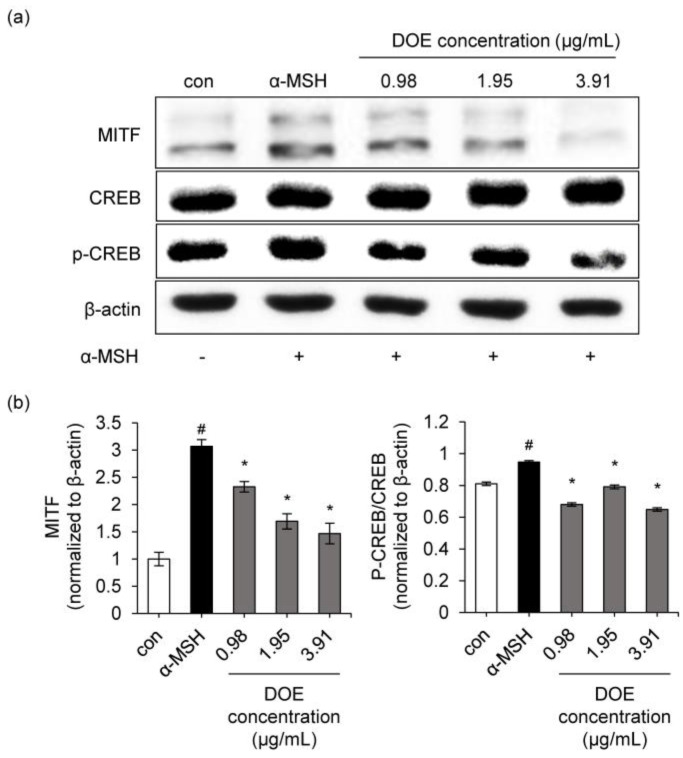
(**a**) Effects of the DOE on the protein expressions of MITF, p-CREB, and CREB in B16F10 cells. B16F10 cells were treated with different concentrations of DOE ranging from 0.98 to 3.91 μg/mL. Protein expressions of MITF, p-CREB, and CREB were analyzed by Western blotting. Equal protein loading was confirmed using β-actin antibody. (**b**) Protein levels of MITF, p-CREB, and CREB proteins. The relative level of each protein was calculated based on the intensity of actin protein and CREB. ^#^ *p* < 0.05 vs. control. * *p* < 0.05 vs. α-MSH-treated group.

MITF is a crucial transcriptional factor and a major regulator of tyrosinase, TRP-1, and TRP-2 expressions in melanogenesis. The negative regulation of MITF leads to inhibition of melanogenesis. The MITF expression of α-MSH group in B16F10 cells increased 3.07-fold compared to the control (Figure 5a). However, 1.95 and 3.91 μg/mL applications of DOE inhibited MITF expressions by 44.9 and 52.2%, respectively, compared to the α-MSH-treated group (Figure 5a,b). Moreover, the extract also significantly blocked the α-MSH-induced phosphorylation of CREB.

### 3.5. Anti-Melanogenic Activity of DOE via Activating ERK/AKT Signaling Pathway

**Figure 6:**
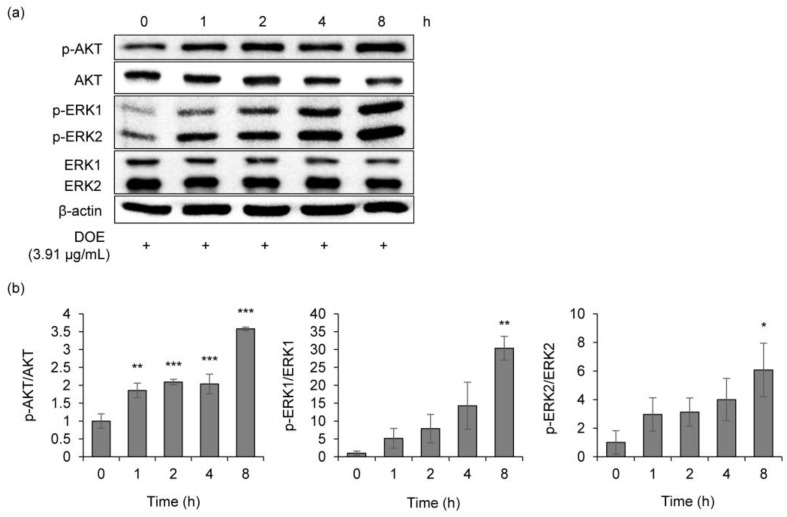
(**a**) Effects of the DOE on the protein expressions of p-AKT, AKT, p-ERK1/2, and ERK1/2 in B16F10 cells. B16F10 cells were treated with 3.91 μg/mL of DOE. Protein expressions of p-AKT, AKT, p-ERK1/2, and ERK1/2 were analyzed by Western blotting. Equal protein loading was confirmed using β-actin antibody. (**b**) Protein levels of p-AKT, AKT, p-ERK1/2, and ERK1/2. The relative level of each protein was calculated based on the intensity of β-actin protein and CREB. * *p* < 0.05, ** *p* < 0.01, and *** *p* < 0.001 vs. 0 h.

The inhibition of the phosphorylation of AKT leads to MITF activation, resulting in melanin synthesis. Thus, the modulation of AKT was investigated to understand whether the extract might inhibit melanogenesis by regulating this signaling pathway. Treatment with DOE markedly increased p-AKT expression in a time-dependent manner (Figure 6), indicating the anti-melanogenesis effect of DOE. 

ERK activation induced the degradation of MITF, which subsequently reduced tyrosinase expression. To further clarify the role of DOE on melanogenesis reduction, phosphorylated ERK protein levels in α-MSH-stimulated B16F10 cells were analyzed. The extract dramatically increased p-ERK1/2 levels in a time-dependent manner compared to the ERK1/2 level.

## 4. Discussion

Great advances have been made in elucidating the molecular and cellular mechanisms underlying melanin biosynthesis process, including melanin formation and melanosome transfer. This has led to the development of a variety of skin-brightening agents that target tyrosinase, tyrosinase-related melanogenic enzymes, and the constituents of the melanogenic signaling pathway: melanocyte homeostasis and melanosome transfer to the keratinocytes. 

The inhibition of tyrosinase, a rate-limiting enzyme in melanin biosynthesis, and the regulation of MITF, a transcriptional factor, are the general approaches for finding anti-melanogenic agents. Arbutin, hydroquinone, kojic acid, and flavonoid derivatives were reported to show anti-melanogenic activity by targeting tyrosinase and MITF [22]. Centaureidin, niacinamide, and PAR-2 inhibitors have been known to interrupt melanosome transfer and down-regulate melanogenesis. In addition, the capability of α-hydroxy acids, salicylic acid, and linoleic acid to accelerate epidermal turnover and desquamation could result in skin brightening [23]. Interestingly, some compounds, such as acetylsalicylic acid, exhibit their hypopigmentation effects via inhibiting tyrosinase gene expression, although they do not exert any direct suppressive activity on mushroom tyrosinase activity [11].

Tyrosinase expression is transcriptionally regulated by MITF, which is activated by CREB and inhibited by PI3K/AKT. The activation of the PI3K/AKT pathway down-regulates tyrosinase transcription and melanogenesis in B16F10 cells [24]. The p38-MAPK pathway is known to be associated with the regulation of melanogenesis. Moreover, CREB is a crucial regulator of MITF. Phosphorylated CREB activates MITF, which binds to the promoter of target genes in melanogenesis. Thus, it is important to verify whether the DOE activates or inhibits AKT, CREB, and p38 pathways. 

In this investigation, DOE reduced in vitro and cellular tyrosinase activity. Moreover, melanin biosynthesis was also reduced in a concentration-dependent manner without cytotoxicity in the range of concentrations tested (0.98 to 3.91 μg/mL) in B16F10 cells, and the extent of inhibition was comparable to that of arbutin at 50 μg/mL. A significant correlation of in vitro activity of mushroom tyrosinase with the cellular activity of tyrosinase and melanin biosynthesis was not found in melanoma cells, as reported by using kojic acid and several herbal extracts [25]. However, the *Daphne odora* extract inhibited in vitro and cellular tyrosinase and reduced α-MSH-induced melanin contents in mouse B16F10 cells, showing superior and efficient depigmenting effect. In addition, the usefulness of DOE in human skin could be verified after application to the normal human melanocytes. 

α-MSH enhances MITF expression, modulating the expression of melanogenic enzymes, such as TRP-1, TRP-2, and tyrosinase, which eventually trigger melanin biosynthesis. Thus, it was important to demonstrate the anti-melanogenic activity of the extract by verifying the down-regulation of tyrosinase and TRP expression. The extract significantly inhibited TRP-1, TRP-2, and tyrosinase protein levels in α-MSH-stimulated B16F10 cells. In addition, the results are also consistent with the ability of the extract to inhibit α-MSH-enhanced MITF expression, suggesting that DOE inhibits α-MSH-triggered melanogenesis at the transcriptional level. 

*Daphne odora*, belonging to the red bean flower family, is characterized by the presence of daphnetin, daphnin, daphnodorin, umbelliferone, (+)-pinoresinol, (−)-lariciresinol, larieiresinol, (−)-secoisolariciresinol, matairesinol, and (−)-wikstromol [26]. In this investigation, daphnetin, one of the constituents of *Daphne odora*, accounted for 0.047% of DOE, and effectively inhibited in vitro tyrosinase activity. Recently, the anti-melanogenic effect of daphnetin was exerted by the regulation of the PKA/CREB and the ERK/MSK1/CREB pathways [27]. Daphnetin has been known to possess notable therapeutic properties against malaria, allergy, inflammatory disorders, and malignant tumors [28,29,30]. In addition, (−)-lariciresinol, (+)-pinoresinol, and (−)-wikstromol are known to be potential MITF inhibitors [31]. Therefore, DOE may have strong anti-melanogenesis activity due to the presence of several constituents of melanogenesis inhibitors, including daphnetin, although we do not know the exact identity of the constituent that exerts the anti-melanogenic effect, and it is not clear whether the constituents exhibit the anti-melanogenic effect individually or collectively [32].

Taken together, the results from this study indicate that *Daphne odora* extracts significantly increased the expression of phosphorylated AKT and ERK, while it reduced the activation of CREB and MITF. DOE-induced AKT/ERK, and CREB/MITF expression might play a pivotal role in anti-melanogenesis in B16F10 cells by *Daphne odora*. These results also suggest that the DOE could potentially be developed as a depigmenting agent.

## 5. Conclusions

Our data demonstrate that DOE exerts anti-melanogenic activities that may be attributed to the inhibition of tyrosinase and TRPs via the suppression of MITF/CREB and the activation of ERK/AKT pathways, resulting in anti-melanogenesis.

## Data Availability

Not applicable.
